# A species-specific miRNA participates in biomineralization by targeting CDS regions of Prisilkin-39 and ACCBP in *Pinctada fucata*

**DOI:** 10.1038/s41598-020-65708-4

**Published:** 2020-06-02

**Authors:** Xuejing Zhu, Yan Chen, Zhen Zhang, Shuyan Zhao, Liping Xie, Rongqing Zhang

**Affiliations:** 1 The Ministry of Education Key Laboratory of Protein Sciences, School of Life Sciences, Tsinghua University, Beijing, 100084 China; 2 Zhejiang Provincial Key Laboratory of Applied Enzymology, Yangtze Delta Region Institute of Tsinghua University, 705 Yatai Road, Jiaxing, 314006 China; 3 College of Biological, Chemical Sciences and Engineering, Jiaxing University, Jiaxing, 314001 China

**Keywords:** Molecular biology, Zoology

## Abstract

Biomineralization is a sophisticated biological process precisely regulated by multiple molecules and pathways. Accumulating miRNAs have been identified in invertebrates but their functions in biomineralization are poorly studied. Here, an oyster species-specific miRNA, novel_miR_1 was found to regulate biomineralization in *Pinctada fucata*. Target prediction showed that novel_miR_1 could target Prisilkin-39 and ACCBP by binding to their coding sequences (CDS). Tissue distribution analysis revealed that the expression level of novel_miR_1 was highest in the mantle, which was a key tissue participating in biomineralization. Gain-of-function assay *in vivo* showed that biomineralization-related genes including Prisilkin-39 and ACCBP were down-regulated and shell inner surfaces of both prismatic and nacreous layer were disrupted after the over-expression of novel_miR_1, indicating its dual roles in biomineralization. Furthermore, the shell notching results indicated that novel_miR_1 was involved in shell regeneration. Dual-luciferase reporter assay *in vitro* demonstrated that novel_miR_1 directly suppressed Prisilkin-39 and ACCBP genes by binding to the CDS regions. Taken together, these results suggest that novel_miR_1 is a direct negative regulator to Prisilkin-39 and ACCBP and plays an indispensable and important role in biomineralization in both prismatic and nacreous layer of *P. fucata*.

## Introduction

Biomineralization is a process during which inorganic ions are orderly deposited under biological regulation of biomacromolecules^1,2^. Due to the excellent mechanical properties, medical and commercial values of shells and pearls, biomineralization in mollusks has attracted great attention. The formation of pearls and shells is precisely regulated by shell matrix proteins (SMPs) that account for less than 5% of the shell weight but are considered as the main component regulating biomineralization process^3–5^. Though SMPs involved in biomineralization have been extensively studied^6–8^, little is known about the regulatory mechanism of their gene expression.

microRNAs (miRNAs) are an important class of endogenous single-stranded non-coding RNAs (~22nt) that can regulate gene expression at the post-transcriptional level by repressing translation or inducing degradation of mRNAs^9^. miRNAs can be divided into two types, conserved miRNAs and novel species-specific miRNAs based on their evolutionary conservation^10^. Conserved miRNAs keep the sequence conserved in the course of evolution and share similar functions with homologues across species^11,12^. In contrast, novel miRNAs are species-specific with no homologues found across species, which might dedicate crucially to adaptation against environment changes^13,14^. miRNAs interact with target genes by binding to the 3’ untranslated region (3’UTR) or the coding sequence (CDS) of mRNAs. Most miRNAs identified in animals were reported to target genes by binding to the 3’UTR^15^. Accumulating evidence supports that miRNAs can also target genes via the CDS in animals^16–18^. However, there are still few reports illustrating miRNAs binding to the CDS in invertebrates.

miRNAs have been proved indispensable in almost all biological processes^19^, including biomineralization. It has been found that miRNAs play an important role in bone and teeth biomineralization in vertebrates^20,21^. For instance, miR-182 was found to negatively regulate osteoblast proliferation and differentiation by suppressing a transcription factor FoxO1^22^. miR-145 and miR-143 were reported to participate in tooth development by regulating odontoblast differentiation^23^. In addition, miR‐150 was found to stimulate osteoblast function and promote osteoblast mineralization for bone formation by negatively regulating MMP14^24^. In recent years, more and more miRNAs have been identified in invertebrates^25^. In bivalve mollusks, miRNAs have been identified from oyster *Pinctada fucata*^26,27^, scallop *Chlamys farreri*^28^, oyster *Crassostrea gigas*^29^, clam *Tegillarca granosa*^30^, oyster *Crassostrea hongkongensis*^31^ and oyster *Ostrea edulis*^32^. Majority of studies about invertebrate miRNAs have focused on immune modulation^33–35^, less is known about their modulation in biomineralization. Based on the significance of miRNAs in regulation of gene expression, more research on the biomineralization-related miRNAs is needed to explore their roles in shell formation.

*Pinctada fucata* (*P. fucata*) is an ideal model organism for studying biomineralization, from which researchers have identified a great number of SMPs^7,8^ including Prisilkin-39^36^ and ACCBP^37^. miRNAs involved in shell formation of this oyster should also be given attention because they can play important roles by modulating the gene expression of SMPs. In previous studies, researchers have obtained a series of miRNAs in *P. fucata* by deep sequencing and computational prediction^26,27,38^. Nevertheless, information concerning the roles of these miRNAs in shell formation is limited. Only three conserved miRNAs in *P. fucata* were reported to participate in nacre formation. Pm-miR-2305 was verified interaction with Pearlin, a nacre formation-related SMP by binding to 3’UTR of the gene^39^. Pm-miR-29a, a homologue of miR-29a regulating bone formation in mammals, was observed modulating biomineralization in the nacreous layer by targeting the neuropeptide Y receptor type 2 (Y2R) via the 3’UTR^40^. And it was found that pm-miR-183 participated in nacre formation by targeting 3’UTR of PmRunt, which was a runt-like transcriptional factor involved in biomineralization possibly through promoting the expression of collagen VI-like and Nacrein^41^. Therefore, little is known about the miRNA-mediated regulation in biomineralization. So far, there has been no report verifying a species-specific miRNA involved in both prismatic and nacreous layer formation by binding to the CDS regions.

With the draft genome released^42^ and transcriptome sequenced^43–45^, the sequence information of plenty of miRNAs have been obtained by small RNA sequencing in *P. fucata* (data not published). Among these, novel_miR_1, a species-specific miRNA, was predicted to target several SMP genes, suggesting its potential roles in modulating biomineralization. The purpose of this study was to verify the hypothesis that novel_miR_1 could regulate biomineralization process and shed light on the mechanism of this miRNA-mediated shell formation. Thus, in the present study, we performed functional assays *in vivo* and *in vitro* to provide insight into how novel_miR_1 was involved in shell biomineralization. We confirmed that novel_miR_1 targeted Prisilkin-39 and ACCBP by binding to the CDS regions. These results provide evidence that novel_miR_1 plays dual roles in biomineralization and contribute to the understanding of mechanism underlying shell formation from a new perspective.

## Results

### Precursor prediction, homology searches and target prediction

**Figure 1:**
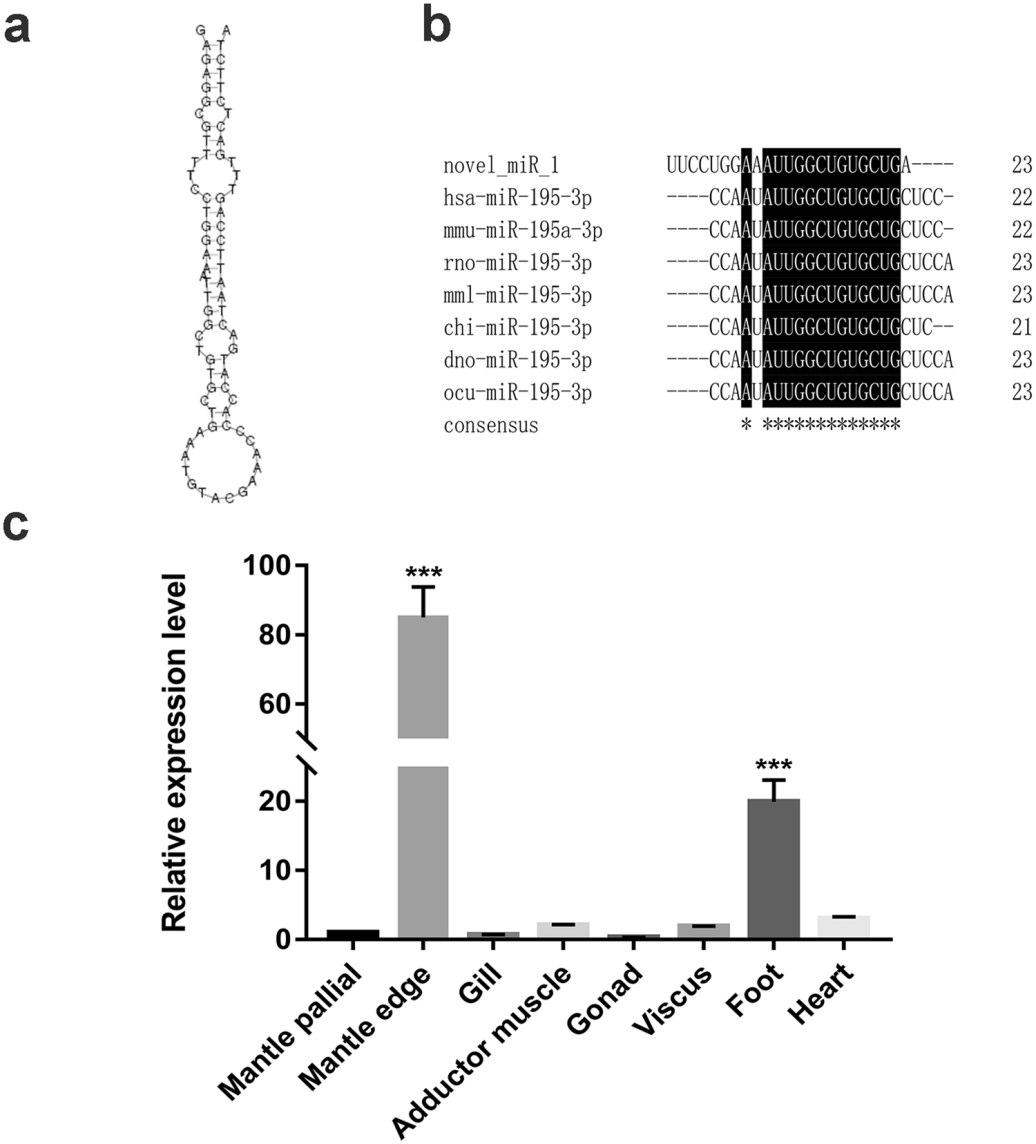
Identification and distribution of novel_miR_1. (**a**) Hairpin structure of the precursor novel_miR_1 analyzed by M-fold. (**b**) Results of homology searches for novel_miR_1. The results showed that no homologues were found in miRBase 22.1. Nucleotide similarity could be found in miR-195-3p from *Homo sapiens* (hsa), *Mus musculus* (mmu), *Rattus norvegicus* (rno), *Macaca mulatta* (mml), *Capra hircus* (chi), *Dasypus novemcinctus* (dno), *Oryctolagus cuniculus* (ocu). The conserved nucleotides were written in white on a black background and identical nucleic acid was marked with “*”. (**c**) The relative expression levels of novel_miR_1 in different tissues by qRT-PCR (From left to right: mantle pallial, mantle edge, gill, adductor muscle, gonad, viscus, foot, heart). The expression level of novel_miR_1 in the mantle pallial was used as the control. The results were analyzed by one-way ANOVA method.

The sequence of novel_miR_1 identified from small RNA sequencing was used to blast against the transcriptome database of *P. fucata*. Results showed that unigene16271 contained the mature sequence of novel_miR_1. To identify the secondary structure of the precursor novel_miR_1, the frank sequences (nearly 100 bp) next to the mature sequence were predicted by M-fold. Unigene16271 contained a 77 bp harpin structure and the mature miRNA was at the 5’ stem of the hairpin (Fig. 1a). Homology searches showed that no miRNA from other species was found matched with either mature or stem-loop sequence of novel_miR_1 (Fig. 1b). Multi-alignments of novel_miR_1 sequence from *P. fucata* with miR-195-3p sequences from other species (hsa: *Homo sapiens*; mmu: *Mus musculus*; rno: *Rattus norvegicus*; mml: *Macaca mulatta*; chi: *Capra hircus*; dno: *Dasypus novemcinctus*; ocu: *Oryctolagus cuniculus*) were shown (Fig. 1b). To find the potential target genes of novel_miR_1, miRanda software was used to predict the target relationship with 3’UTR and CDS sequences from all known genes of *P. fucata*. Consequently, a total of 25 genes were annotated, including SMP genes such as Prisilkin-39, ACCBP, PfCHS1, PfMG11, N151. Target information was shown in Table 1.

**Table 1:** Target genes of novel_miR_1 by miRanda prediction.

Gene ID	Gene Annotation	Protein Name	Score	Energy
GI_ 334350827	Prisilkin-39 like	*pfu* Prisilkin-39	164	−26.59
GI_ 93359257	ACCBP1	*pfu* ACCBP	159	−20.94
GI_ 152205943	PfCHS1 like	*pfu* PfCHS1	160	−24.6
GI_71063304	PFMG11A	*pfu* PFMG11	164	−24.89
GI_269935951	N151	*pfu* N151	156	−21.44
GI_63273	Collagen XII	*gga* Collagen XII	159	−27.01
GI_206597436	Collagen alpha-1	*gga* Collagen alpha-1(III) chain precursor	159	−21.13
GI_60302772	Myosin regulatory light chain interacting protein	*gga* E3 ubiquitin-protein ligase	160	−21.64
GI_47550941	Calmodulin-binding carboxy-terminal kinesin	*spu* Kinesin-C	168	−20.95
GI_45384490	Collagen alpha-1(XIV) chain precursor	*gga* Collagen alpha-1(XIV) chain	152	−23.11
GI_45384318	Collagen alpha-1(XII) chain precursor	*gga* Collagen alpha-1(XII) chain	164	−20.78
GI_4519619	Collagen pro alpha-chain	*hdi* Collagen pro alpha-chain	156	−22.5
GI_9229910	Myosin	*mye* Myosin	158	−21.3
GI_29378343	Calcium/calmodulin-dependent serine protein kinase 1	*lst C*alcium/calmodulin-dependent serine protein kinase 1	158	−20.66
GI_157690435	Carbonic anhydrase I	*pmo* Carbonic anhydrase I	167	−25.82
GI_71733128	Myhc4(nonmuscle myosin)	*aca* Nonmuscle myosin II	158	−21.3
GI_327290154	Tubulin beta chain-like	*aca* Tubulin beta-4B chain	177	−33.97
GI_210078005	Activin receptor type2 like	*cgi* Activin type II receptor precursor	152	−20.43
GI_126303629	5’-3’ exoribonuclease 2	*mdo* 5’-3’ exoribonuclease 2 isoform X2	161	−23.16
GI_22859616	FoxA	*pvu* Fork head protein	152	−20.77
GI_347800228	Pif like protein	*mga* BMSP	163	−20.91
GI_353558891	Tyrosinase-like protein 12	*pma* Tyrosinase-2	158	−20.2
GI_4115773	Paramyosin	*mga* Paramyosin	165	−20.86
GI_320090050	Catalase	*pfu* Catalase	166	−21.04
GI_219806593	Myosin regulatory light chain	*cgi* Tropomyosin	155	−24.66

### Tissue distribution of novel_miR_1 in *P. fucata*

qRT-PCR was performed to detect the expression levels of novel_miR_1 in different tissues. The results showed that novel_miR_1 was widely distributed in all tissues tested and the expression of novel_miR_1 in the mantle edge was much higher than in other tissues, suggesting it participated in biomineralization (Fig. 1c).

### Gain-of-function assay of novel_miR_1 *in vivo*

**Figure 2:**
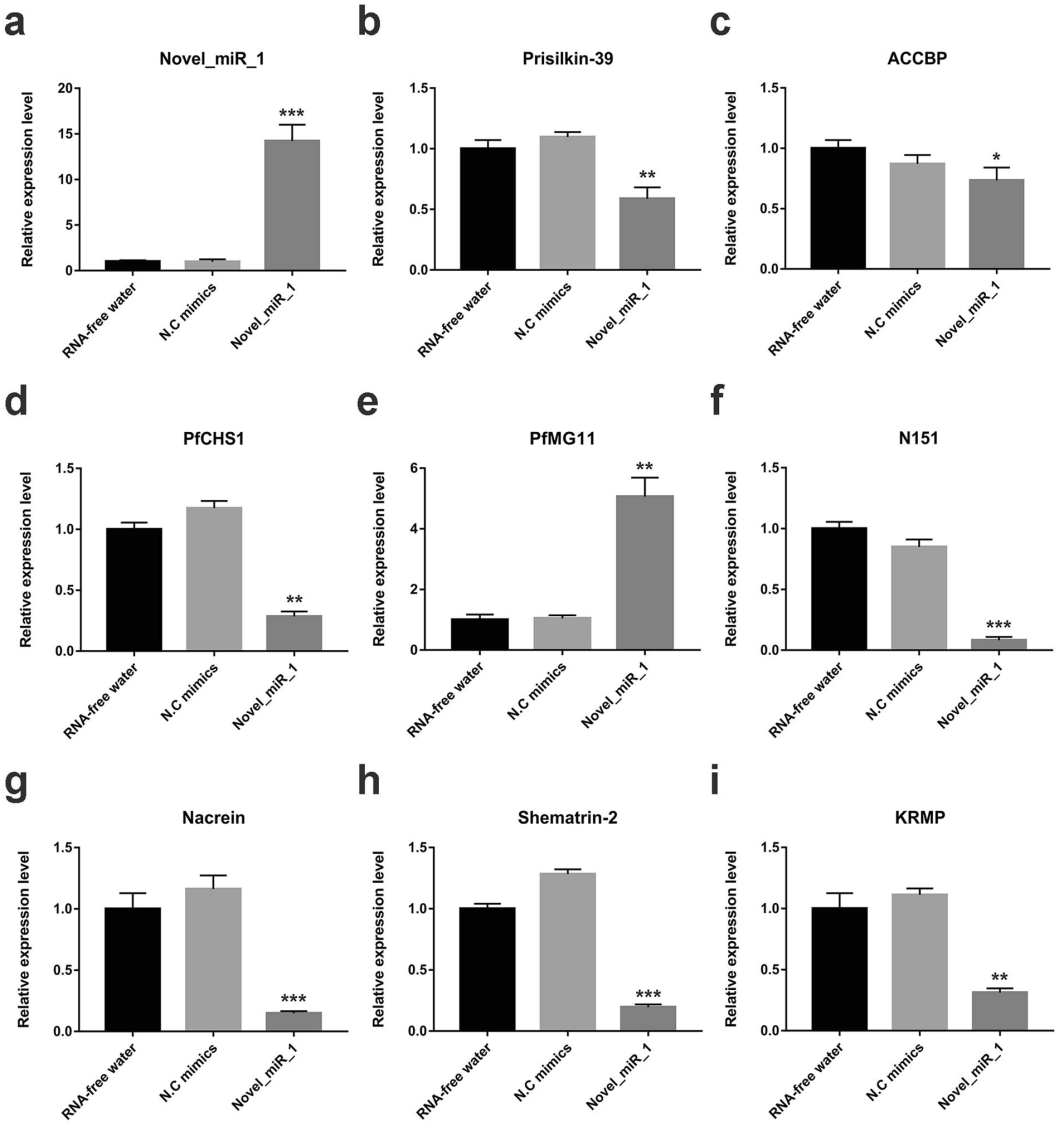
Gain-of-function assay of novel_miR_1 *in vivo*. Gain-of-function assay was conducted *in vivo* by injection of novel_miR_1 mimics. At 3d post injection, qRT-PCR was performed to evaluate the expression levels of novel_miR_1, putative target genes and several biomineralization-related genes in mantle tissues. (**a–i**) Relative expression level of novel_miR_1, Prisilkin-39, ACCBP, PfCHS1, PfMG11, N151, Nacrein, Shematrin-2, KRMP. The RNA-free water group was used as control. The results were analyzed by Student’s T test.

To investigate roles of novel_miR_1 in shell formation, we conducted gain-of-function assay of novel_miR_1 *in vivo* by injecting novel_miR_1 mimics into the adductor muscle. RNase-free water and NC mimics were also injected into the adductor muscle in another two group of oysters respectively. qRT-PCR was performed to detect the expression level of novel_miR_1 in the mantle at 3d post the injection. The group injected with RNase-free water (RNA-free water group) was considered as the control. As shown in Fig. 2a–i, no significant change was observed in the expression levels of novel_miR_1 and related genes in the group injected with N.C mimics (N.C group) compared to the RNA-free water group. As shown in Fig. 2a, in the group injected with novel_miR_1 mimics (novel_miR_1 group), the expression level of novel_miR_1 increased to 14-fold compared with the RNA-free water group (p < 0.001), which gave evidence of successful over-expression of novel_miR_1 *in vivo*. Meanwhile, we measured the expression levels of putative target SMP genes, including Prisilkin-39, ACCBP, PfCHS1, N151, PfMG11 in the mantle after the injection. Compared to the RNA-free water group, the expression levels of Prisilkin-39, ACCBP, PfCHS1, N151 genes in the novel_miR_1 group decreased by 41%, 27%, 72%, and 92% respectively (Fig. 2b,c,d,f). However, the expression of PfMG11 was upregulated to 5-fold of the control (Fig. 2e). To further investigate the effects of novel_miR_1 on the biomineralization-related genes, we measured the gene expression of Nacrein, which is a nacre formation-related SMP, and two prismatic layer formation-related SMPs, Shematrin-2 and KRMP. As expected, they were all significantly inhibited (p < 0.01) (Fig. 2g–i), indicating the participation of novel_miR_1 in the formation of both the nacreous layer and the prismatic layer.

**Figure 3:**
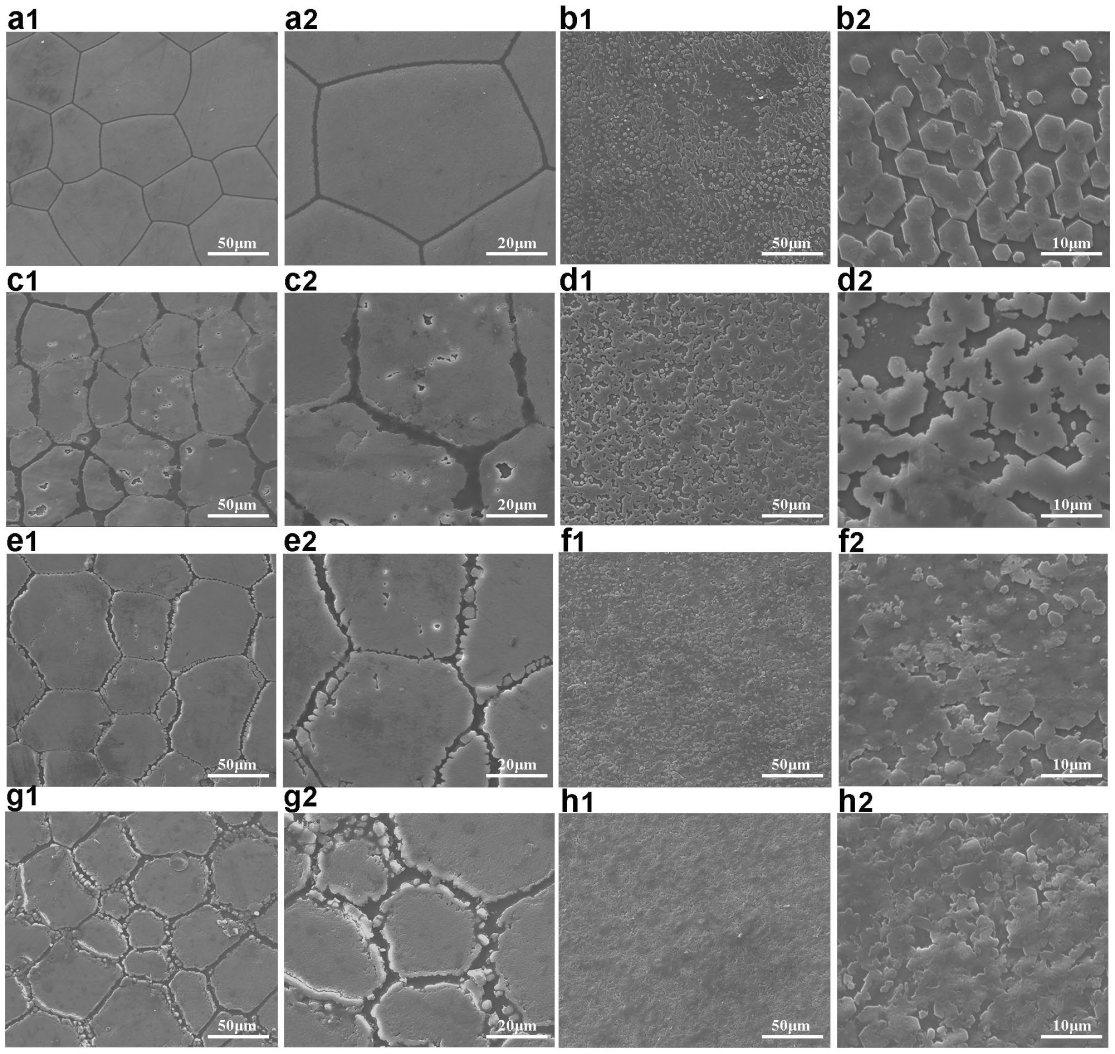
The scanning electron microscope images of the ultrastructure of shell inner surfaces. (**a1**) SEM images of the prismatic layer for the oysters injected with N.C mimics. The surface was smooth with clearly visible edges between adjacent polygons. **(b1**) SEM images of the nacreous layer for the oysters injected with N.C mimics. The layer showed a stair-like growth pattern with rectangular or hexagonal tablets. (**c1,e1,g1**) SEM images of the prismatic layer for the oysters injected with novel_miR_1 mimcs. The layer showed disordered growth in different degrees. (**d1,f1,h1**) SEM images of the nacreous layer for the oysters injected with novel_miR_1 mimics. The layer showed overgrowth of aragonite with irregular tablets. (**a2,b2,c2,d2,e2,f2,g2,h2**) Enlarged images of (**a1,b1,c1,d1,e1,f1,g1,h1**), respectively. Scale bars: 50 μm in (**a1,b1,c1,d1,e1,f1,g1,h1**); 20 μm for high-magnification images in (**a2,c2,e2,g2**); 10 μm for high-magnification images in (**b2,d2,f2,h2**).

The dual roles of novel_miR_1 in shell biomineralization were further confirmed by observing the inner surfaces of the shells in gain-of-function assay using SEM. We scanned across the growing edge and the regions near the growing edge of the shells were observed. In the N.C mimic-injected group, smooth surface with clearly visible edges between regular polygons was seen in the prismatic layer (Fig. 3a1,a2) and the nacreous layer showed a stair-like growth pattern with rectangular or hexagonal flat tablets on the surface (Fig. 3b1,b2), which was the same morphology seen in the untreated pearl oysters. After injecting novel_miR_1 mimics, the growth of both the prismatic and nacreous layer was disturbed and showed abnormal ultrastructure in different degrees of disruption. The surface of the prismatic layer became rough with small holes and the organic frameworks vanished gradually (Fig. 3c1,c2). The prisms showed obvious jagged edges and some edge regions of polygons were broken (Fig. 3e1,e2). The prismatic layer showed disordered organic frameworks, where the lacuna between adjacent polygons became larger and irregular crystals emerged (Fig. 3g1,g2). For the nacreous layer, the rectangular or hexagonal tables became irregular (Fig. 3d1,d2). The original nacre tablets were covered with randomly accumulated crystals (Fig. 3f1,f2). Thickened irregular tablets were fused together, making the boundaries obscure (Fig. 3h1,h2). All SEM images of the both layers for each of the five oysters injected with novel_miR_1 mimics were shown in Figs. S1 and S2.

### Expression patterns of novel_miR_1 and target genes during shell regeneration

**Figure 4:**
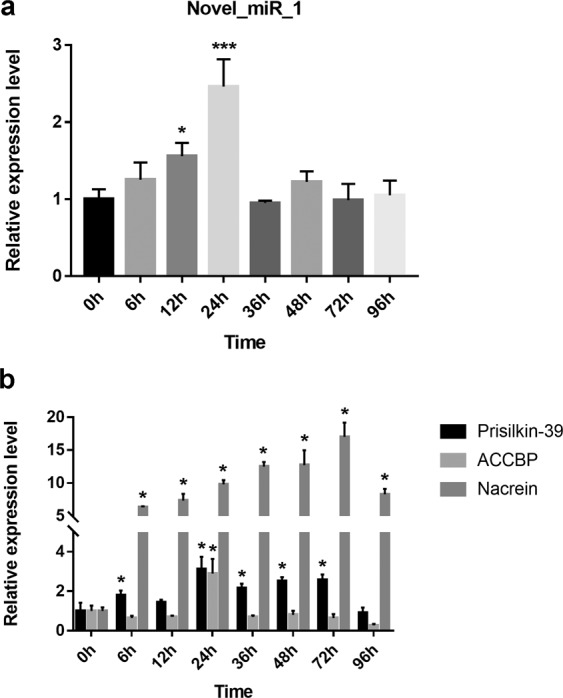
Expression levels during shell repairing. The relative expression levels of novel_miR_1, Prisilkin-39, ACCBP and Nacrein in the mantle of *P. fucata* during shell repairing after shell notching. The expression levels at 0 h were considered as controls. Differences were assessed by one-way ANOVA.

To reveal potential functions of novel_miR_1 in biomineralization, we performed shell notching assay at different time points. During shell regeneration, expression levels of novel_miR_1 and several biomineralization-related genes in the mantle, which had the highest novel_miR_1 expression among various tissues, were measured by qRT-PCR. Nacrein was detected as positive control. One-way ANOVA method was used to measure the significance of the difference at each time point. As shown in Fig. 4a, novel_miR_1 expression increased to reach the peak within 0 h to 24 h after shell notching. The maximum was approximately 2.5-fold of the control. After 24 h, the expression level decreased rapidly and in the following hours, the expression retained at the same level as the control with slightly fluctuating. ACCBP had a similar pattern to that of novel-miR_1, with the expression peak (2.9-fold of the control) appearing at 24 h (Fig. 4b). Prisilkin-39 increased from 0 h to 24 h with slightly fluctuating and reached the maximum (3-fold of the control) at 24 h, then decreased slowly thereafter, and finally was down-regulated to the basal level of the control (0.91-fold of that in control group) at 96 h (Fig. 4b). Nacrein expression increased dramatically from 0 h to 6 h and maintained at a high level until 96 h (Fig. 4b).

### Target verification of novel_miR_1 *in vitro*

**Figure 5:**
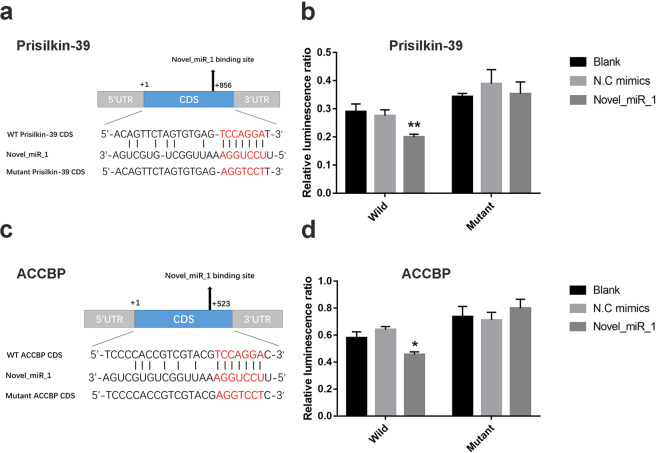
Interactions between novel_miR_1 and target genes *in vitro*. (**a**) Putative binding sites of novel_miR_1 on the CDS region of Prisilkin-39 predicted by miRanda and mutations were written in red. (**b**) The relative luminescence ratio in Prisilkin-39 CDS luciferase reporter assay. (**c**) Putative binding sites of novel_miR_1 on the CDS region of ACCBP predicted by miRanda and mutations were written in red. (**d**) The relative luminescence ratio in ACCBP CDS luciferase reporter assay. Blank groups were used as controls. The results were analyzed by Student’s T test.

Prisilkin-39 and ACCBP were chosen to investigate their interactions with novel_miR_1 *in vivo* because putative binding sites of novel_miR_1 were found on the CDS regions of their genes respectively. To further validate the prediction, we cloned the coding sequences into the pmirGLO vector to construct a recombinant vector (designated as wild type vector) and mutations were made on the CDS seed sequences to construct another vector (designated as mutant type vector) (Fig. 5a,c). Dual Luciferase reporter assays were performed using recombinant vector carrying either the wild or mutated CDS of the target gene in HEK293T cells co-transfected with N.C mimics (N.C group) or novel_miR_1 mimics (novel_miR_1 group). Cells transfected with merely recombinant vectors served as blank control (blank group). At 48 h post transfection, for Prisilkin-39 wild type vector, the relative luminescence ratio in the novel_miR_1 group decreased significantly to 69% in comparison with that in blank group (Fig. 5b). For ACCBP wild type vector, an obvious decrease of relative luminescence ratio was likewise seen in the novel_miR_1 group in comparison with that in blank group (79% of the control) (Fig. 5d). Meanwhile, for both Prisilkin-39 mutant type vector and ACCBP mutant type vector, no significant changes were observed in the novel_miR_1 group compared to blank group (Fig. 5b,d). In addition, the relative luminescence ratios in all N.C groups were similar to that in their corresponding blank groups.

## Discussion

Biomineralization is a complex, precise process regulated by multiple molecules^1,2^. However, recent studies have mainly focused on the functions of SMPs in shell biomineralization^8,46–48^, leading to the lack of studies on the regulation of SMPs at upstream transcriptional level. miRNAs were found to regulate the gene expression post-transcriptionally in various biological processes^19^. The miRNAs related to shell biomineralization have not been fully clarified. The main purpose of this study was to verify the hypothesis that novel_miR_1 participated in the regulation of shell formation in *P. fucata*.

In the present study, we obtained the sequence of novel_miR_1, a new species-specific miRNA via small RNA deep sequencing (data not published). Homology searches showed that no homologues of novel_miR_1 across species were found (Fig. 1b). miRNAs are continuously being added to animal genomes through time, and once integrated into gene regulatory networks, they show very rare mutations in the primary sequences^12,49^. Evolutionary conserved miRNAs retain similarity in both sequences and functions across species^11^. miRNAs are instrumental in the evolution of organismal complexity^14^, as new miRNA families emerge to adapt against environment changes^11,13^. Contrary to conserved ones, species-specific miRNAs with no homologues found across species are called novel miRNAs. Previous studies about biomineralization-related miRNAs verified in *P. fucata* just focused on conserved miRNAs^39–41^. In fact, novel miRNAs might play an indispensable role in modulating unique biological processes because it is very likely that species acquire specific functions accompanied by the emergence of new miRNAs. Therefore, we speculated that novel_miR_1 acted irreplaceably in shell biomineralization due to its specificity for the oyster.

The distribution in different tissues indicated that mantle tissues had the highest expression level of novel_miR_1 (Fig. 1c). As is known, the mantle is the most important tissue involved in shell biomineralization from which a mass of SMPs are secreted. The mantle edge is responsible for the formation of the periostracum and the prismatic layer, whereas the mantle pallial is concerned with the formation of the nacreous layer^50^. Considering abundant distribution of novel_miR_1 in the mantle edge, it was speculated that this miRNA-mediated regulation mainly involved in the prismatic layer formation.

Furthermore, target prediction revealed that novel_miR_1 could target multiple genes, indicating its participation in various biological processes (Table 1). Among these genes, SMP genes occupied a large proportion, suggesting that novel_miR_1 might play an important role in regulating biomineralization. Putative binding sites were found between novel_miR_1 and the CDS regions of Prisilkin-39 and ACCBP, which were two SMPs identified as key participants in prismatic and nacreous layer formation respectively. Many previous studies on the regulation of miRNAs have mainly focused on repressing gene expression by binding to the 3’UTR^15^. It has been proved that miRNAs can induce mRNA degradation by binding to 3’UTR^51^, whereas inhibit translation by binding to CDS^17,18^. However, there have been few reports illustrating the miRNA-mediated regulation by binding to CDS of the gene in invertebrates. Our study would provide new evidence for miRNA-mediated modulation by binding to the CDS in invertebrates.

To determine whether novel_miR_1 inhibited gene expression and regulated shell biomineralization, we conducted gain-of-function assay *in vivo*. After the over-expression of novel_miR_1, qRT-PCR was conducted to measure expression levels of five target genes, Prisilkin-39, ACCBP, PfCHS1, PfMG11 and N151. Prisilkin-39 is involved both in the chitinous framework building and in crystal growth regulation by inhibiting aragonite precipitation during the prismatic layer mineralization^36^. ACCBP can control the morphology of nacre tablets in the nacreous layer and regulate the formation of amorphous calcium carbonate (ACC), which functions as a precursor for calcium carbonate biominerals^37^. PfCHS1 is chitin synthase found in both layers, contributing to the formation of the framework for calcification in the shell^52^. PfMG11^53^ and N151^43,54^ are biomineralization-related proteins but have not yet been identified. Typical SMPs, Nacrein^55^, Shematrin-2^56^, and KRMP^46^ were also chosen for investigation. Nacrein, highly expressed in genes related to nacre formation, can modify nacre lamellae morphology by inhibiting aragonite precipitation or growth. Shematrin-2 and KRMP, highly expressed in genes related to prism formation, can function as framework proteins and control calcite formation in the prismatic layer. Given that these genes are important regulators of biomineralization, the change of their expression can suggest the mechanism at molecule level for the morphology change of both layers after the over-expression of novel_miR_1. The results showed that most genes were significantly down-regulated after the over-expression of novel_miR_1 (Fig. 2b,d,f,g–i). We speculated that Prisilkin-39, PfCHS1, N151 were effectively inhibited by novel_miR_1 directly targeting putative binding regions whereas Nacrein, Shematrin-2 and KRMP, on which binding sites of the miRNA couldn’t be found, were more likely to be modulated indirectly by a complicated miRNA-mediated network. In addition, ACCBP was slightly decreased (Fig. 2c), indicating that it could be regulated by multiple molecules or pathways. Unexpectedly, a significant increase was observed in PfMG11 expression (Fig. 2e). As we know, majority of miRNAs were reported to repress gene expression via inhibiting translation or increasing degradation of mRNAs^57^. It is not a common way for miRNAs to promote gene expression post-transcriptionally and the underlying mechanism remains largely unknown^58^. The molecular mechanism of miRNA-mediated regulation on PfMG11 needs further investigation.

To further demonstrate novel_miR-1 regulation on shell formation, the inner surfaces of shells were investigated in gain-of-function assay *in vivo*. Under the observation of SEM, the growth of both prismatic and nacreous layer was disrupted in oysters injected with novel_miR_1 mimics (Fig. 3c1–h2) compared to the normal ultrastructure in the N. C group (Fig. 3a1–b2). The prismatic layer showed disordered formation of prisms in different degrees of disruption (Fig. 3c1-2,e1-2,g1-2), which might be attributed to the decreased expression levels of the prismatic layer formation-related SMPs (Prisilkin-39, PfCHS1, Shematrin-2, KRMP) caused by novel_miR_1 over-expression (Fig. 2b,d,h,i). After over-expression of novel_miR_1, the nacre tablets became irregular (Fig. 3d1,d2), which was similar to that observed in pearl oysters after directly inhibition of ACCBP by injecting anti-body in Ma’s study^59^. Overgrowth of the aragonite crystals disturbed the star-like growth pattern of the nacreous layer (Fig. 3f1-2,h1-2), which was similar to Prisilkin-39 inhibition experiment results by injecting anti-body in Kong’s work^36^. This data was consistent with the decreased expression levels of Prisilkin-39 and ACCBP induced by novel_miR_1 verified in our molecular biology work (Fig. 2b,c). The results above confirmed that novel_miR_1 played dual roles in shell formation.

Shell notching was then performed to survey the expression pattern during shell regeneration. Prisilkin-39 and ACCBP were of interest for further study because each of them was predicted to have a binding site with novel_miR_1 in the protein-coding sequence and they were well-studied as important participants in prismatic and nacreous layer formation respectively. Nacrein, detected in shell notching experiments in previous study^47^, was chosen as positive control. The expression of novel_miR_1 increased at 6 h after shell notching, reaching the peak at 24 h, and then decreased to the basal level in following hours (Fig. 4a). It may be caused by stimulation from injury that induced an immune response. Previous studies have reported that the mantle participates in immunity^60^ and shell repair^48^, and may respond rapidly to injury. A similar pattern was observed for ACCBP (Fig. 4b), indicating that ACCBP might increase due to immune response and then be down-regulated by the peak expression of novel_miR_1 in a regulation delay pattern. Prisilkin-39 expression experienced a process of rising and falling with some fluctuation (Fig. 4b). After rising to reach a peak at 24 h, Prisilkin-39 was down-regulated in a postponed expression pattern possibly caused by the increased miRNAs at the early stage. Though falling, the expression level was still more than 2-fold of the basal level, indicating that Prisilkin-39 expression was the modulating result of other unknown molecules and pathways except novel_miR_1 during shell regeneration. In support of above results, it was speculated that novel_miR_1 was involved in shell regeneration and regulated the gene expression of Prisilkin-39 and ACCBP.

To further verify interactions between novel_miR_1 and the two target genes *in vitro*, dual luciferase reporter assay was carried out. In the present study, results showed that the relative luminescence ratios of Prisilkin-39 and ACCBP wild type vector were decreased remarkably in novel_miR_1 group compared to blank group (Fig. 5b,d), demonstrating that Prisilkin-39 and ACCBP were inhibited by novel_miR_1 by directly binding to their CDS regions. Both mutant type vectors were not influenced by novel_miR_1, which verified specificity of the interactions. Based on above results, Prisilkin-39 and ACCBP were confirmed to be the direct target gene of novel_miR_1. To date, majority of animal miRNAs could regulate genes at post transcriptional level by binding to the target mRNA at 3’UTR regions^15^ and few of them were reported to modulate genes by binding to CDS regions^16,17^. Previous studies reported that miRNAs binding to the CDS mainly led to translation inhibition^17,18^. In this study, verified modulation of novel_miR_1 on Prisilkin-39 and ACCBP by binding to the CDS regions provides new evidence for this uncommon mechanism.

Functional assays verified the effects of novel_miR_1 on the prismatic layer formation-related SMPs (Prisilkin-39, PfCHS1, Shematrin-2, KRMP) (Figs. 2, 4 and 5) and detected the morphology changes of prisms (Fig. 3), which deciphered the potential mechanism of this miRNA-mediated regulation in the prismatic layer formation. This data was in agreement with the significantly higher expression level of novel_mir_1 in the mantle edge (Fig. 1c), highlighting the involvement of this miRNA in the formation of the prismatic layer. From the target prediction results (Table 1) and functional assays (Figs. 2–5),we deduced that novel_miR_1 also had the capacity to affect the nacreous layer formation possibly by binding with putative target SMPs (Prisilkin-39, ACCBP, Pif-like protein) responsible for the nacre formation or indirectly suppressing SMP gene (Nacrein) via middle regulatory factors or signaling pathways. However, the expression level of novel_miR_1 in the mantle pallial showed no discrepancy with that of the tissues which were not considered as key organs for biomineralization (Fig. 1c), indicating that it might not function as key participants in the nacreous layer formation for adult oysters. The synthesis of novel_miR_1 was not activated in the mantle pallial, possibly due to the regulatory roles of upstream molecules or signaling pathways on the precursor gene of this miRNA. Further investigations are needed to elucidate the upstream regulatory mechanism of the biosynthesis of novel_miR_1.

## Conclusion

In summary, our results show that a newly identified species-specific miRNA, novel_miR_1 plays dual roles in biomineralization. Functional assays *in vivo* and *in vitro* provide evidence that novel-miR_1 participates in shell formation by regulating biomineralization-related genes and the CDS regions of Prisilkin-39 and ACCBP are direct targets of novel_miR_1. This study provides a novel understanding of the miRNA-mediated regulation in biomineralization in mollusks. Moreover, it lays foundation for future study about the relationship between miRNAs and multiple molecules in the regulatory network and further investigations are needed to understand the regulatory mechanism of novel_miR_1.

## Materials and Methods

### Statement

We confirm that all methods were carried out in accordance with the approved guidelines and regulations. All experiments were approved by the Animal Ethics Committee of Tsinghua University, Beijing, China. The study protocol in the experiments of the animals was approved by the Ethics Committee of National Center for Clinical Laboratories. The 3 R principles about animal experimentation (reduction, replacement, and refinement) were observed strictly.

### Pearl oyster

Adult pearl oysters *P. fucata* (averaging 5–6 cm in shell length and 30–40 g of wet weight) were collected from the Zhanjiang Pearl Farm (Guangdong, China). The oysters were maintained in aerated 20 °C artificial seawater with 3% salinity for two weeks post experiments.

### Bioinformatic analysis

The mature sequence of novel_miR_1 found in the unigene16271 was predicted from small RNA deep sequencing (data not published) and the secondary structure of novel_miR_1 precursor was predicted by M-fold program using the unigene16271 sequence. Homologues of novel_miR_1 were obtained using blastn program at miRBase 22.1 (released in October 2018) (http://www.mirbase.org) using mature and stem-loop sequences of novel_miR_1 and similar sequences were aligned by ClustalX. The target genes of novel_miR_1 were searched globally by miRanda software with 3’UTR and CDS sequences from all oyster genes.

### RNA extraction, cDNA synthesis and qRT-PCR

Total RNA was extracted from the mantle of *P. fucata* using TRIzol reagent (Invitrogen, USA) according to its protocol. The reverse transcription of mRNAs was carried out using PrimeScript RT Master Mix (Perfect Real Time) (Takara, Japan) and the reverse transcription of miRNAs was carried out using Mir-X miRNA First-strand Synthesis kit (Clontech, USA) to obtain cDNA templates for qRT-PCR. The quantitative real-time PCR was carried out using SYBR Premix Ex Taq (Takara, Japan) in a LightCycler 480 system (Roche Diagnostics, Switzerland). Primers used in the present study were listed in Table S1. The relative expression of genes and novel_miR_1 was calculated using ${2}^{-\Delta \Delta Ct}$ method after normalization with GAPDH or U6 snRNA.

### Expression and distribution pattern of novel_miR_1

To analyze the expression patterns of novel_miR_1 in different tissues, total RNAs of the mantle pallial, mantle edge, adductor muscle, gill, gonad, viscus, foot and heart of *P. fucata* were extracted and qRT-PCR was performed using the method described above.

### Gain-of-function assay of novel_miR_1 *in vivo*

Novel_miR_1 mimics and negative control mimics (N.C mimics) were synthesized by GenePharma (Shanghai, China) and diluted in 0.1 μg/μL with RNase-free water. Novel_miR_1 mimics are identical with novel_miR_1 in nucleotide sequence and N.C mimics are negative control that couldn’t mimic any miRNAs and target any oyster genes. A total of 15 oysters were randomly divided into three groups and were injected into the adductor muscle with 100 μL RNase-free water, 100 μL solutions with novel_miR_1 mimics (0.1 μg/μL) or N.C mimics (0.1 μg/μL) respectively. The N.C group served as the negative control. At 3d post injection, total RNAs were extracted from the mantle tissues of each group oysters to detect the expression levels of novel_miR_1 and candidate genes. qRT-PCR was conducted to investigate the over-expression of novel_miR_1 and the effects of gain-of-function on the expressions of the SMP genes. Each trial contains three parallel samples and the experiments were repeated twice.

### SEM observation of shells

To examine the morphology differences of the shell inner surface after over-expression of novel_miR_1, the shells above were then observed by scanning electron microscope (SEM, FEI Quanta 200, Netherlands). First, all shells were collected, cut into small pieces, washed with Milli-Q water and air-dried. Then, cleaned shells were coating with gold for 60 s and subjected to observation by sem.

### Shell notching experiment

Pearl oysters were randomly divided into eight groups with five oyster each. A narrow notch adjacent to the adductor muscle was sawn in the shell of each oyster and mantle issues from five individuals in each group were sampled at 0, 6, 12, 24, 36, 48, 72, 96 h after notching. qRT-PCR was then performed to measure the expression levels of novel_miR_1 and related genes in the mantle extracted at different time point. Each trial contains three parallel samples and the experiments were repeated twice.

### Recombinant vector construction

The complete CDS of Prisilkin-39 or ACCBP, which contained a putative binding site of novel_miR_1, was cloned using gene-specific primers (Table S1) and then inserted into pmirGLO vector (Promega, USA) to obtain the wild type vector. To verify the specificity of the interaction, mutated vectors were obtained via the way that mutations were made in the seed sequences where novel_miR_1 was predicted to be completely complementary with novel_miR_1 on the wild-type vector. All constructs were verified by DNA sequencing.

### Dual-luciferase reporter assay

HEK293T cells were co-transfected with 625 μg recombinant vectors and 25pmol of either novel_miR_1 mimics or N.C mimics. Detailed information of the transfection was listed in Table S2. Cells transfected merely with recombinant vectors were employed as blank group. After harvesting for 48 h, the cells were plated for dual luciferase reporter assay (Promega, USA) using Varioskan Flash (Thermo Scientific, Waltham, MA, USA). Each trial contains three parallel samples and the experiments were repeated twice.

### Statistical analysis

All data obtained was given as means ± SD. Student’s T-test and One-way ANOVA analysis were used by GraphPad Prism7 software. Significant differences among groups were marked with “*” at p < 0.05, “**” at p < 0.01 and “***” at p < 0.001.

## Supplementary information

Supplementary Information.
