# Supplementary material for: A species-specific miRNA participates in biomineralization by targeting CDS regions of Prisilkin-39 and ACCBP in Pinctada fucata
Source: Sci Rep. 2020 Jun 2;10:8971. doi: 10.1038/s41598-020-65708-4 (PMC7265298; doi:10.1038/s41598-020-65708-4)
Supplement: Supplementary file 1 — Supplementary Information. [file 41598_2020_65708_MOESM1_ESM.pdf]

Supplementary information

A species-specific miRNA participates in biomineralization by targeting CDS regions of Prisilkin-39 and ACCBP in *Pinctada fucata*

Xuejing Zhu[1], Yan Chen[1], Zhen Zhang[1,2], Shuyan Zhao[1,2], Liping Xie[1] & Rongqing Zhang[1,2,3]*

1. the Ministry of Education Key Laboratory of Protein Sciences, School of Life Sciences, Tsinghua University, Beijing 100084 China;
2. Zhejiang Provincial Key Laboratory of Applied Enzymology, Yangtze Delta Region Institute of Tsinghua University, 705 Yatai Road, Jiaxing 314006 China;
3. College of Biological, Chemical Sciences and Engineering, Jiaxing University, Jiaxing 314001, China.

*To whom correspondence may be addressed.

E-mail: rqzhang@mail.tsinghua.edu.cn.

Tel: +86-010-62772630.

Table S1. Primers and RNAs

Category	Name	Sequence (5'-3')
RNAs	Novel_mir_1 mimics-S	UUCCUGGAAAUUGGCUGUGCUGA
	Novel_mir_1 mimics-A	AGCACAGCCAAUUUCCAGGAAUU
	N.C mimics-S	UUCUCCGAACGUGUCACGUTT
	N.C mimics-A	ACGUGACACGUUCGGAGAATT
Vector construct primers	Prisilkin39_SacI	CGAGCTCATGAAAGGATTCCTGACGCTCTTG
	Prisilkin-39_XhoI	CCGCTCGAGTTAACAGTCCTTGTGACCATG
	ACCBP_SacI	CGAGCTCCTTGTTCTGTAAACAACAGATGA
	ACCBP_XhoI	CCGCTCGAGTTACTTATCCTTATCGTTATGTTCA
	Prisilkin-39_mutant-F	CATATTACAGTTCTAGTGTGAGTGGTCCATACGGATATTA
	Prisilkin-39_mutant-R	TAATATCCGTATGGACCACTCACACTAGAACTGTAATATG
	ACCBP_mutant-F	TCTACCTCCCCACCGTCGTACGTGGTCCACATGAACTTAA
	ACCBP_mutant-R	TTAAGTTCATGTGGACCACGTACGACGGTGGGGAGGTAGA
qRT-PCR primers	Novel_miR_1-F	TTCCTGGAAATTGGCTGTGCTGA
	GAPDH-F	TATTTCTGCACCGTCTGCTG
	GAPDH-R	ATCTTGGCGAGTGGAGCTAA
	Prisilkin-39-F	TGGGTATTGTCAAAGTCGTC
	Prisilkin-39-R	CAACTCCTACTGGGAAACCT
	ACCBP-F	CTCATCACTCCTTCTGTTCT
	ACCBP-R	GCCTTACTTATCCTTATCGT
	PfCHS1-F	GTCCTATCAGCCTGTTACCC
	PfCHS1-R	GTGCCTCATCTTGTTCTTGTCC
	PfMG11-F	ACAGGTATCGTTGTTGGTAG
	PfMG11-R	CAGTTTCGTATTTTGGTGGA
	N151-F	TAAAGGGAAGACAGAGGCAG
	N151-R	GCAGGAGAGGTAATTTGGGA
	Nacrein-F	GGCTTTGGCGACGAACCGGA
	Nacrein-R	ACACGGGGGAGTGGTCAGGG
	Shematrin-2-F	CCCCGGTAGAGTGTCTGGTA
	Shematrin-2-R	AGTTCCGACGGATACACCTG
	KRMP-F	AAGAAATGTCACCCTTGGGATTGG
	KRMP-R	AATCATCGCCACCATATCCATCG

Table S2. Group and transfection information of the dual luciferase reporter assay.

Group	Blank group	N.C mimics group	Novel_miR_1 group
Recombined vector	625ng	625ng	625ng
N.C mimics		25pmol	
Novel_miR_1 mimics			25pmol

A total of 625ng recombined plasmid containing either wild type or mutant type Prisilkin-39 or ACCBP CDS was transfected into cells of blank group. An additional of 25pmol N.C mimics or novel_miR_1 mimics were co-transfected with the plasmid into cells of N.C mimics group and novel_miR_1 group, respectively.

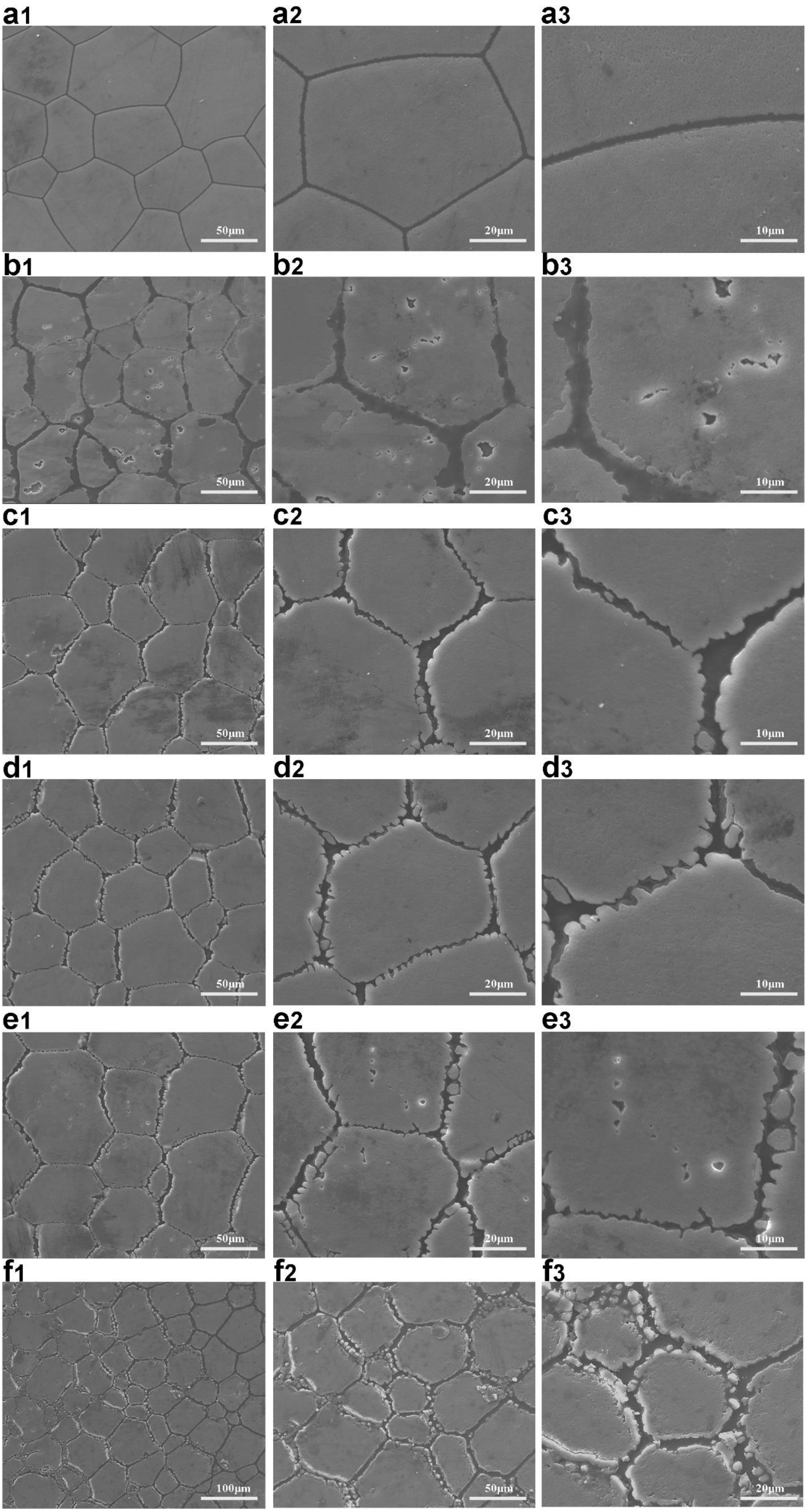
a1
50μm
a2
20μm
a3
10μm
b1
50μm
b2
20μm
b3
10μm
c1
50μm
c2
20μm
c3
10μm
d1
50μm
d2
20μm
d3
10μm
e1
50μm
e2
20μm
e3
10μm
f1
100μm
f2
50μm
f3
20μm

Figure S1. SEM images of the prismatic layer for the five oysters injected with novel_miR_1 mimics. (a1) SEM images of the prismatic layer for the oysters injected with N.C mimics. The surface of the prisms was smooth with clearly visible edges between adjacent regular polygons. (b1, c1, d1, e1, f1) SEM images of the prismatic layer for each of the five oysters. The prismatic layer showed disordered growth in different degrees. (a2, b2, c2, d2, e2, f2) Enlarged images of a1, b1, c1, d1, e1, f1, respectively. (a3, b3, c3, d3, e3, f3) Enlarged images of a2, b2, c2, d2, e2, f2, respectively. Scale bars: 50μm in a1, b1, c1, d1, e1, f2; 100 μm in f1; 20μm in a2, b2, c2, d2, e2, f3; 10μm in a3, b3, c3, d3, e3;

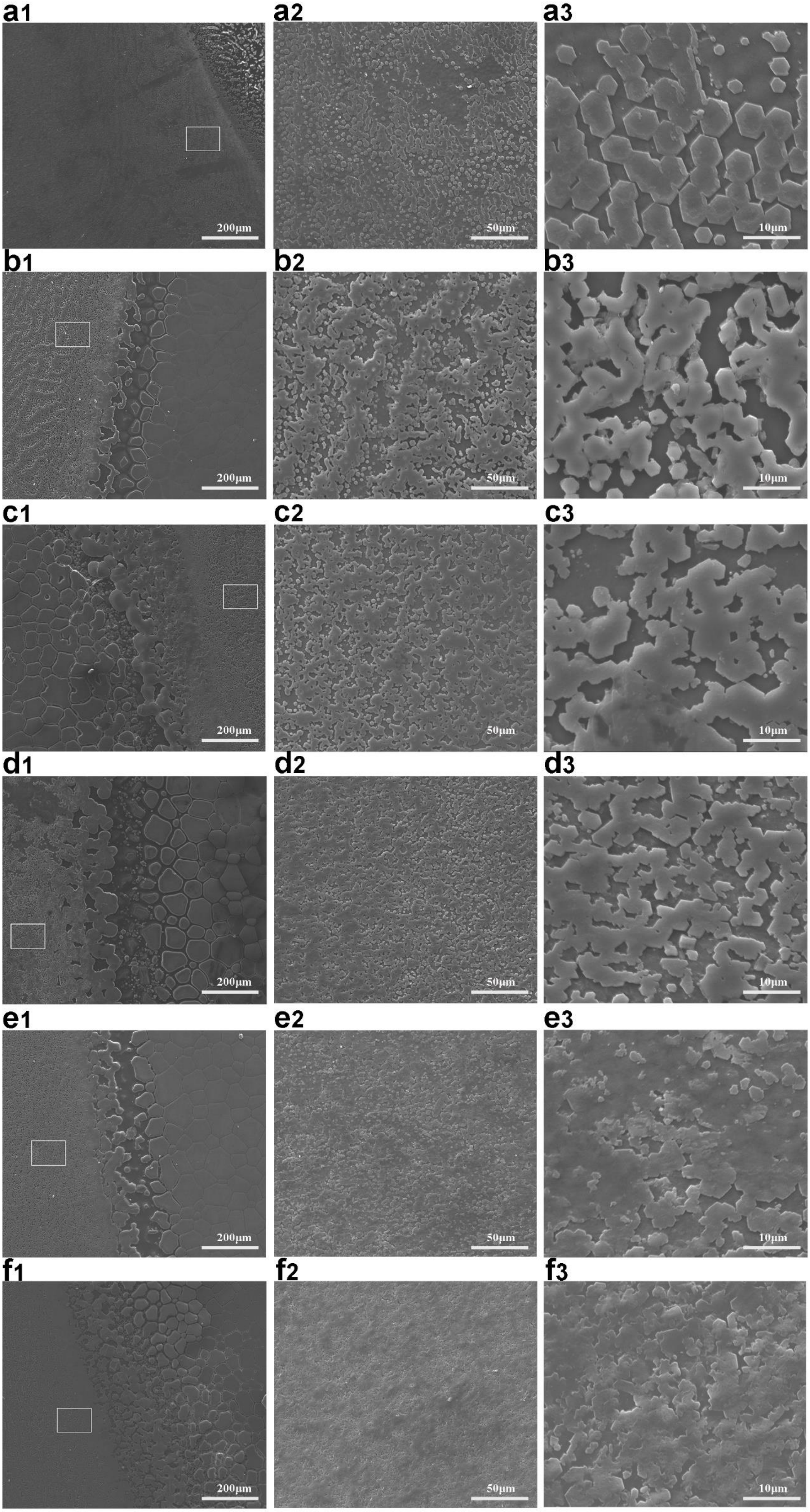
a1
a2
a3
b1
b2
b3
c1
c2
c3
d1
d2
d3
e1
e2
e3
f1
f2
f3
200μm
50μm
10μm

Figure S2. SEM images of the nacreous layer for the five oysters injected with novel_miR_1 mimics. (a1) SEM images of the nacreous layer for the oysters injected with N.C mimics. The observed region was near the growing edge. (a2) Enlargement of the box in a1. The nacreous layer showed a stair-like growth pattern with rectangular or hexagonal tablets. (b1, c1, d1, e1, f1) SEM images of the nacreous layer for each of the five oysters. The observed regions were near the growing edge. (b2, c2, d2, e2, f2) Enlargement of the box in b1, c1, d1, e1, f1, respectively. The nacreous layer showed overgrowth of aragonite in different degrees. (a3, b3, c3, d3, e3, f3) Enlarged images of a2, b2, c2, d2, e2, f2, respectively. Scale bars: 200μm in a1, b1, c1, d1, e1, f1; 50μm in a2, b2, c2, d2, e2, f2; 10μm in a3, b3, c3, d3, e3, f3;
